# Role of β1 integrin in thrombocytopoiesis

**DOI:** 10.12703/r/10-68

**Published:** 2021-09-01

**Authors:** Maria Mazzarini, Paola Verachi, Fabrizio Martelli, Anna Rita Migliaccio

**Affiliations:** 1 Biomedical and Neuromotor Sciences, Alma Mater University Bologna, Italy; 2 National Center for Preclinical and Clinical Research and Evaluation of Pharmaceutical Drugs, Rome, Italy; 3 University Campus Biomedico, Rome, Italy; 4 Myeloproliferative Neoplasm-Research Consortium, New York, NY, USA

**Keywords:** β1 integrin, thrombocytopoiesis

## Abstract

Thrombocytopoiesis is a complex process beginning at the level of hematopoietic stem cells, which ultimately generate megakaryocytes, large marrow cells with a distinctive morphology, and then, through a process of terminal maturation, megakaryocytes shed thousands of platelets into the circulation. This process is controlled by intrinsic and extrinsic factors. Emerging data indicate that an important intrinsic control on the late stages of thrombopoiesis is exerted by integrins, a family of transmembrane receptors composed of one α and one β subunit. One β subunit expressed by megakaryocytes is the β1 integrin, the role of which in the regulation of platelet formation is beginning to be clarified. Here, we review recent data indicating that activation of β1 integrin by outside-in and inside-out signaling regulates the interaction of megakaryocytes with the endosteal niche, which triggers their maturation, while its inactivation by galactosylation determines the migration of these cells to the perivascular niche, where they complete their terminal maturation and release platelets in the bloodstream. Furthermore, β1 integrin mediates the activation of transforming growth factor β (TGF-β), a protein produced by megakaryocytes that may act in an autocrine fashion to halt their maturation and affect the composition of their surrounding extracellular matrix. These findings suggest that β1 integrin could be a therapeutic target for inherited and acquired disorders of platelet production.

## Introduction

**Figure 1.**
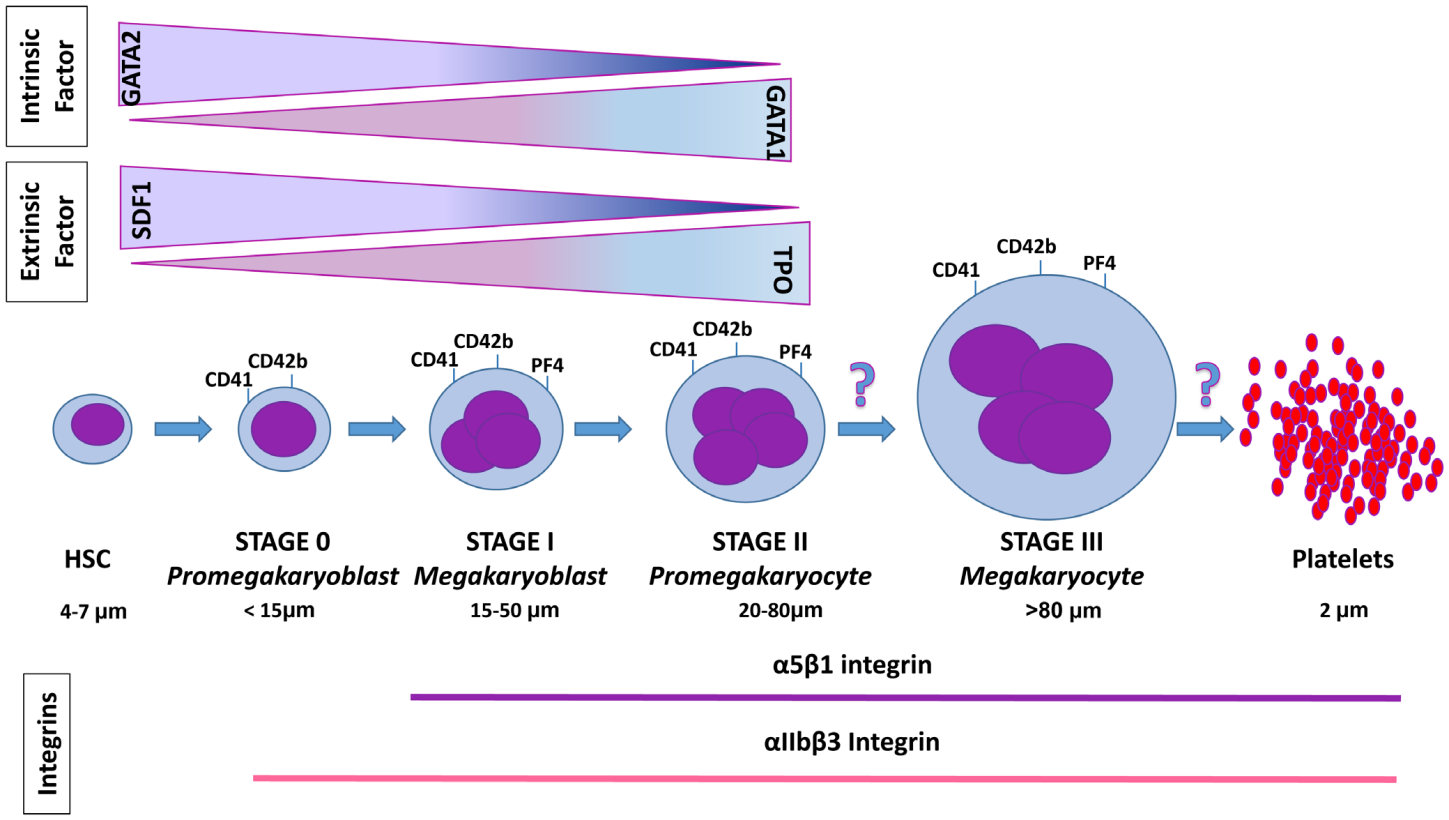
Diagram of megakaryocytopoiesis and of its terminal phase, thrombopoiesis. Megakaryocytes (MKs) are generated from the hematopoietic stem cells (HSCs) under the control of intrinsic factors, the most important of which are GATA1 and GATA2, and extrinsic factors, thrombopoietin (TPO) and stromal cell–derived factor 1 (SDF-1). Based on distinctive morphological markers, MK precursors are divided into four classes of progressively more mature cells: the promegakaryoblast (stage 0), the megakaryoblast (stage I), the promegakaryocyte (stage II), and finally the mature MK (stage III), which is capable of releasing platelets. The maturation of the MKs is also defined by the specific cell surface markers CD41 (integrin-α2b), CD42b (platelet glycoprotein 1b α-chain), and platelet factor 4 (PF4). The pattern of expression of the integrins α5β1 and αIIbβ3 during terminal MK maturation is also indicated. Whereas the factors regulating the progression from HSCs to stage II MKs are known, those leading the terminal maturation of MKs into platelets are still poorly defined. Novel data discussed in this article indicate that one of these factors is represented by α5β1.

Megakaryocytes (MKs) are large cells produced in the bone marrow that undergo a process of terminal maturation to produce platelets, which are blood cells critical for vascular integrity and the trigger of the coagulation process. MKs are produced in the endosteal niche of the bone marrow through a process termed megakaryocytopoiesis, which begins with the hematopoietic stem cells (HSCs) and involves the generation of progenitor cells progressively more committed toward the MK lineage (Figure 1). These progenitor cells eventually generate immature MKs that migrate to the perivascular niche to complete their terminal maturation and to release platelets in the bloodstream. Alternatively, a small fraction of these immature MKs are released directly into the bloodstream to reach the perivascular niche of other organs, such as the lung and the vascular niche of the brain, where they mature and release platelets on demand^1,2^. The process of terminal MK maturation and platelet release is termed thrombocytopoiesis. These processes are finely regulated by a series of extrinsic and intrinsic factors identified through loss- and gain-of-function experiments in animal models and validated by the phenotype of patients carrying relevant genetic mutations. The fact that deregulation of megakaryocytopoiesis is associated with numerous inherited (for example, congenital amegakaryocytic thrombocytopenia^3,4^) and acquired (for example, megakaryoblastic leukemia and primary myelofibrosis^5–7^) MK disorders highlights the importance of deepening our understanding of the mechanisms that regulate platelet production in order to design novel diagnostic, prognostic, and therapeutic tools for these disorders. Here, we review recent information on the role played by integrins, a potentially druggable class of regulators that affect terminal MK maturation and platelet production, and discuss their potential clinical implications.

## Regulation of megakaryocyte commitment

HSC commitment toward the MK lineage is finely regulated by intrinsic and extrinsic factors. The most important intrinsic factors are the transcription factors *GATA2*, which guides commitment of HSCs into MK-restricted progenitor cells, and *GATA1*, which regulates terminal MK maturation (Figure 1)^8–11^. Genetic alterations of *GATA1* are found in several inherited conditions associated with thrombocytopenia and in acute myeloblastic leukemias with a megakaryocyte phenotype (AMKL)^4^. *GATA1* regulates the expression of all of the megakaryocytic-specific genes identified at present, including platelet factor 4 (PF4), and the α chain of glycoprotein IIB (CD41 or *GPIIBα*), expressed in MKs and platelets that enable platelet aggregation by binding fibrinogen and von Willebrand factor (vWF), the initial step of the coagulation cascade^12–14^.

Thrombopoietin (TPO) is a 70-kDa glycoprotein hormone that is the most important extrinsic factor that regulates MK production^15^. TPO is produced primarily by the liver and acts by binding to the TPO receptor (TPO-R, also known as MPL) present on the plasma membrane of HSCs, MK progenitors, and their progeny (platelets)^16,17^. In addition, in bone marrow, osteoblasts can produce TPO, increasing its microenvironmental bioavailability on demand^16^. TPO binding to MPL induces receptor dimerization and activates specific signal transduction pathways, the first element of which is the tyrosine kinase JAK2^17^. Once phosphorylated, JAK2 migrates to the nucleus to activate the expression of MK-specific genes. Activated JAK2 also phosphorylates the cytoplasmic domain of the TPO-R, which serves as a scaffold on which a number of secondary signaling molecules are attracted and phosphorylated, such as STAT5, altering their function^17^. The effects of TPO on thrombopoiesis can be augmented by additional growth factors, the most important of which are interleukin 6 (IL-6), also produced by the liver, and IL-3 and granulocyte-macrophage colony-stimulating factor (GM-CSF), produced by cells of the bone marrow microenvironment^18–21^.

Commitment of HSCs into MK-restricted progenitor cells occurs in the proximity of the endosteal niche within the trabecular bone where these cells are retained by the interaction between the CXCR4 receptor and CXCL12 (previously termed stromal cell–derived factor 1 [SDF-1] produced by the cells of the niche^22^). To drive the migration of MKs from the endosteal to the perivascular niche, required for normal MK maturation, the expression of CXCR4 is progressively downregulated, and although CXCR4 is still detectable on mature MKs and platelets, these cells are unable to respond to CXCL12 stimulation^23^.

In spite of the strong *in vitro* and *in vivo* evidence supporting the primary regulatory role exerted by TPO on thrombopoiesis, the clinical experience with native TPO for the treatment of thrombocytopenias was abandoned because of an immune response. In its place, both peptide and organic molecule TPO-R agonists (TRAs) (for example, romiplostim and eltrombopag) have shown efficacy in rescuing the HSC defects and restoring hemopoiesis in patients with aplastic anemia and in promoting thrombopoiesis in a number of settings, either directly or indirectly^24^.

## Regulation of terminal megakaryocyte maturation and biogenesis of platelets

On the basis of morphological markers identified in 1975 by Dr. Dorothea Zucker Franklin^25,26^, the cellular stages leading to terminal MK maturation are divided into four classes: (1) promegakaryoblasts (*<*15 μm in diameter without morphological hallmarks); (2) megakayoblasts, cells larger in size (15–50 μm in diameter, stage I of MK maturation), characterized by a cytoplasm rich in ribosomes bound to well-developed rough endoplasmic reticulum and a large nucleus which express lineage-specific markers, such as vWF and CD41^27^ (Figure 1). Megakaryoblasts duplicate their DNA but do not undergo cell division, a process that results in endoduplication, leading to progressive polyploidization, which characterizes the terminal maturation of this lineage^28^. (3) Promegakaryocytes (stage II) have grown to the size of mature MKs (about 80 μm in diameter) and start to display the demarcation membrane system (DMS), the massive invagination of the plasma membrane which compartmentalizes the cytoplasm of the cells, encasing their granules into distinctive zones defined as platelet territories^29^. (4) Mature MKs (stage III) display a polylobated nuclei and a mature DMS with distinctive platelet territories and express the surface markers CD42b and CD61^8,30^.

One of the important steps of the terminal maturation is the organization of the α and dense granules, endosomic vesicles containing MK-specific growth factors, adhesion receptors, and coagulations factors, into compartmentalized areas delineated by the DMS, the platelet territories^29^. The α-granules (about 500 nm in size) are divided into stimulatory and inhibitory granules that exert, respectively, a positive or a negative control on angiogenesis, tissue repair, and osteogenesis. The stimulatory granules contain vascular endothelial growth factor (VEGF), angiopoietin 1 (AGN1), fibroblast growth factors (FGFs), transforming growth factor-β (TGF-β), osteoprotegerin (OPG), and bone morphogenetic protein 4 (BMP4), whereas the inhibitory granules contain endostatin, thrombospondin, and vWF^29^. The dense granules (about 300 nm in size) contain small molecules such as ADP, serotonin, and epinephrine, responsible for initiating and augmenting the coagulation cascade^29^.

Through endoduplication, mature MKs feature a polyploid karyotype with up to 64N sets of chromosomes (average number 16N)^31^. This process is due, at least in part, to altered localization of a Ras homolog family member A (RhoA), which precludes completion of the contractile ring and spindle elongation that are required for the dissociation of the sister chromosomes at the end of the metaphase plates^32^. The process of endomitosis (chromosome duplication without cell division) results in the great size which characterizes mature MKs, allowing the cells to produce high numbers of proplatelets and platelets. Sixteen N MKs produce, on average, up to about 2000 platelets each^28,31^.

Platelets are small (about 2 μm) cytoplasm fragments enclosed in a membrane rim released by the MKs into the bloodstream, and the events leading to their release are emerging. Mature MKs anchor themselves to the endothelium of the sinusoids of the bone marrow, extending their cytoplasmic protrusions into the bloodstream (Figure 1). *In vitro*, proplatelet elongation is driven by dynein-dependent sliding of overlapping cortical microtubule bundles, while *in vivo* fusion between the internal and the plasma membrane of the MKs drives the large protrusions to extend themselves into the sinusoidal space for the release of platelets^2,33^. Using *in vitro* modelling, Ito and colleagues identified that this process is regulated by growth factor insulin binding protein 2 (IGFBP2), macrophage migration inhibitory factor (MIF), and nardilysin (NRDC)^2^. IGFBP2 and MIF promote the anchoring of the MKs to the endothelium while NRDC, a zinc-dependent endopeptidase, favors the elongation of the proplatelets in the bloodstream, by interacting with HDAC6, and in platelet shedding, by interacting with α and β1 tubulin^2^. Platelet shedding is also directly regulated by physical challenges (turbulence, flow speed, and shear forces) of the bloodstream, which activate the heavy chain 9 of myosin IIα, a protein encoded by *MYH9*, and megakaryoblastic leukemia 1 (*MKL1*), an element of the mechano-transduction pathway^2,34^. These physical stimuli also activate calcium fluxes through the cation channel transient receptor subfamily V member 4 (TRPV4). Increased calcium flux in turn activates β1 integrin, increasing the crosstalk of the MKs with collagen and other components of the extracellular matrix (ECM), leading to AKT (also known as protein kinase B) phosphorylation, promoting platelet spreading, thrombus growth, and clot retraction^35,36^.

As mentioned earlier, although stage III MKs and platelets express MPL, the function of the TPO/MPL axes in terminal MK maturation is unclear^37^. Accumulating evidence indicates that, by contrast with the commitment process, terminal MK maturation and platelet production are MPL-independent^38^. The observation that, once bound to MPL, the TPO/MPL complex is internalized and destroyed by the lysosome machinery suggests that expression of MPL on platelets is a feedback mechanism to restrain the plasma concentration of TPO in response to platelet number rather than a regulator of platelet production^39,40^. The factors that regulate the last phase of terminal MK maturation have been unknown for a long time. As discussed below, new information indicates that, in addition to platelet shedding, β1 integrin is an important regulator of the terminal MK maturation process.

## The role of integrins in thrombopoiesis

Integrins are a large family of heterodimeric transmembrane proteins that regulate tissue architecture by establishing cell–cell and cell–ECM interactions^41^. These proteins contain a large extracellular domain and a smaller intracellular domain linked by a transmembrane-spanning region. The active integrin complex is an obligatory heterodimer of two subunits: the α and the β subunit. In mammals, the α subunit is encoded by 18 different genes whereas the β subunit is encoded by eight different genes^42,43^. Since the expression of these different genes is activated in lineage-specific fashions, each cell type expresses a defined combination of α and β subunits. This combinatorial heterogeneity allows the great level of plasticity that maintains the anatomic organization of the different tissues.

**Figure 2.**
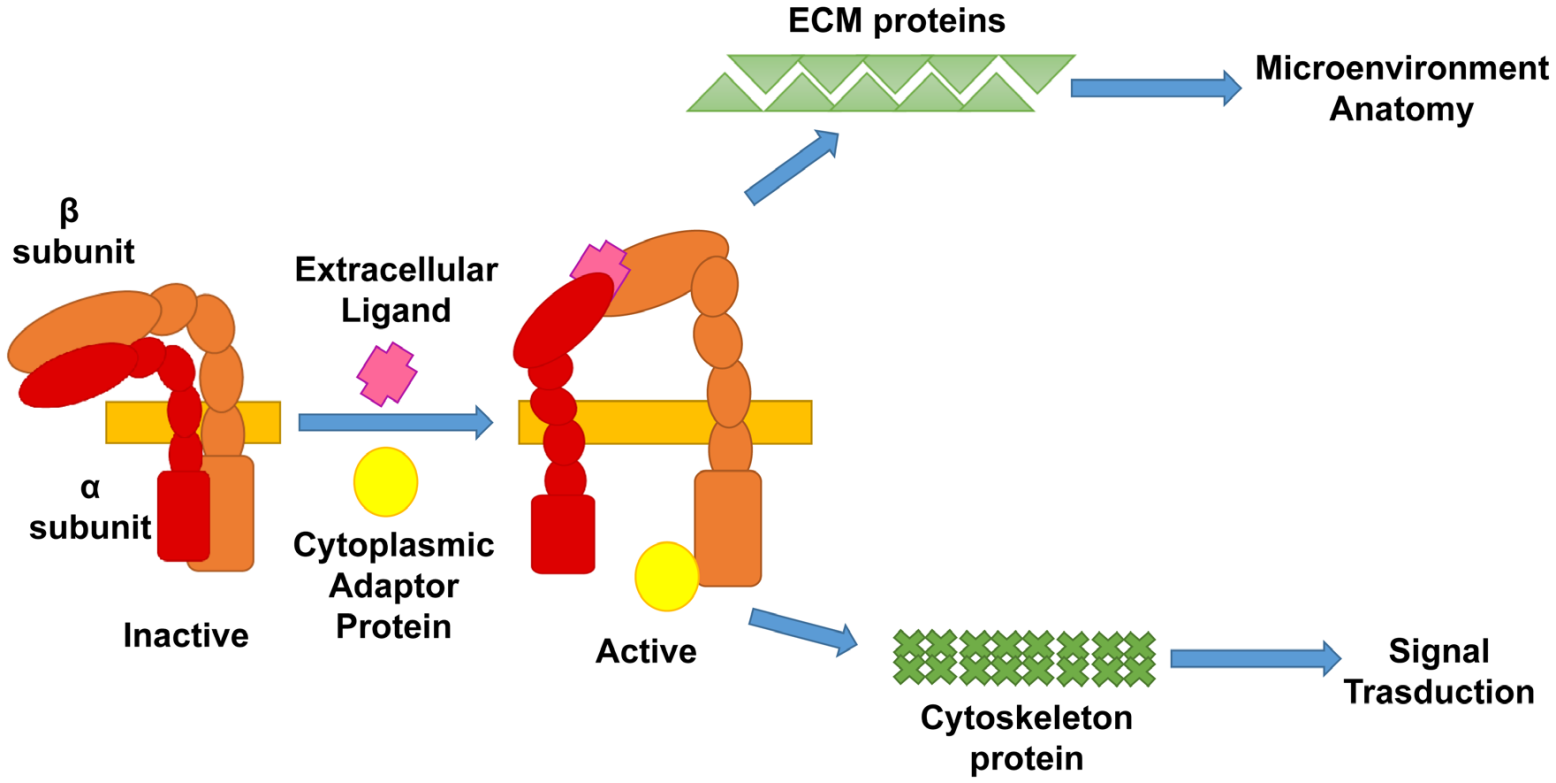
Diagram depicting the structure of the integrins. Integrins are obligate heterodimer transmembrane receptors composed by an α (red) and β (orange) subunit. Integrins are activated by binding to either extracellular ligands or cytoplasmic adaptor proteins (talin and kindlin). Binding of this heterodimer to its ligands induces a conformational change that activates the receptor allowing to bind either proteins of the extracellular matrix (ECM), such as fibronectin, and/or to protein of the cytoskeleton (actin) through the FAK (focal adhesion kinase) and the Rho pathway. Binding of the integrin complex to the ECM regulates the spatial organization of the cells within the tissues while that to the cytoskeleton sends mechano-transduction signal which activates gene transcription or platelets release or both.

Integrins regulate interactions both among different cells and among the cells and the ECM and are activated by outside-in or inside-out (talins or kindlins, also defined as cytoplasmic adaptor proteins) ligands or both^44^. Once bound to its ligand, the integrin complex undergoes a conformational change that allows, on one hand, the pocket formed by the extracellular domains of the two subunits to bind proteins of the ECM and, on the other, the intracellular domain of the β subunit to bind the focal adhesion kinase (FAK) (Figure 2). Binding to the ECM provides the signal that localizes the cells within the microenvironment and contributes to the organization of the overall architecture of a tissue. Binding to FAK mediates binding of the integrin complex to the cytoskeleton and sustains cell survival, proliferation, and polarity^41,44^. Intracellular signaling in MKs is indirectly potentiated by TPO by increasing FAK content^45^. These proteins have numerous pleiotropic functions, and their alterations are implicated in the dysregulation of tissue homeostasis in many diseases, including cancer. (See “Role of β1 integrin alterations in the etiology of diseases associated with altered megakaryocytopoiesis, including cancer” below.)

Although alteration of talin expression in MKs alters platelet functions in Wistar Furth rats, possibly by impairing the formation of their dense granules^46–48^, the phenotype of talin 1^49^ or talin 2^50^ does not include evident platelet disfunctions, indicating that these adaptors are unlikely to be involved in the regulation of platelet formation.

The integrin complexes most expressed by the MKs are the αVβ3^51^, α3β1, and α5β1^52^. These complexes, when activated, are involved in the control of the adhesion of the cells with the endosteal niche and in migration^53,54^. Although the biochemical details of the effects of αVβ3 on MK maturation are still lacking, the clinical importance of this complex is highlighted by the fact that autoantibodies against αVβ3 are responsible for some forms of idiopathic autoimmune thrombocytopenia^51^. More information is available on the mechanisms used by the α5β1 complex to regulate MK adhesion and migration. The β1 subunit of this complex (encoded by the *ITGB1* gene) cooperates with the α5 subunit in anchoring MKs to matrix fibronectin and—by inducing dynamin 2- and 3-dependent CXCR4 down-modulation, on one hand, and cytoskeletal changes and pseudopod formation, on the other—allows the MKs to detach from the endosteal niche and to migrate along the fibronectin fibers through the microenvironment^53,54^.

**Figure 3.**
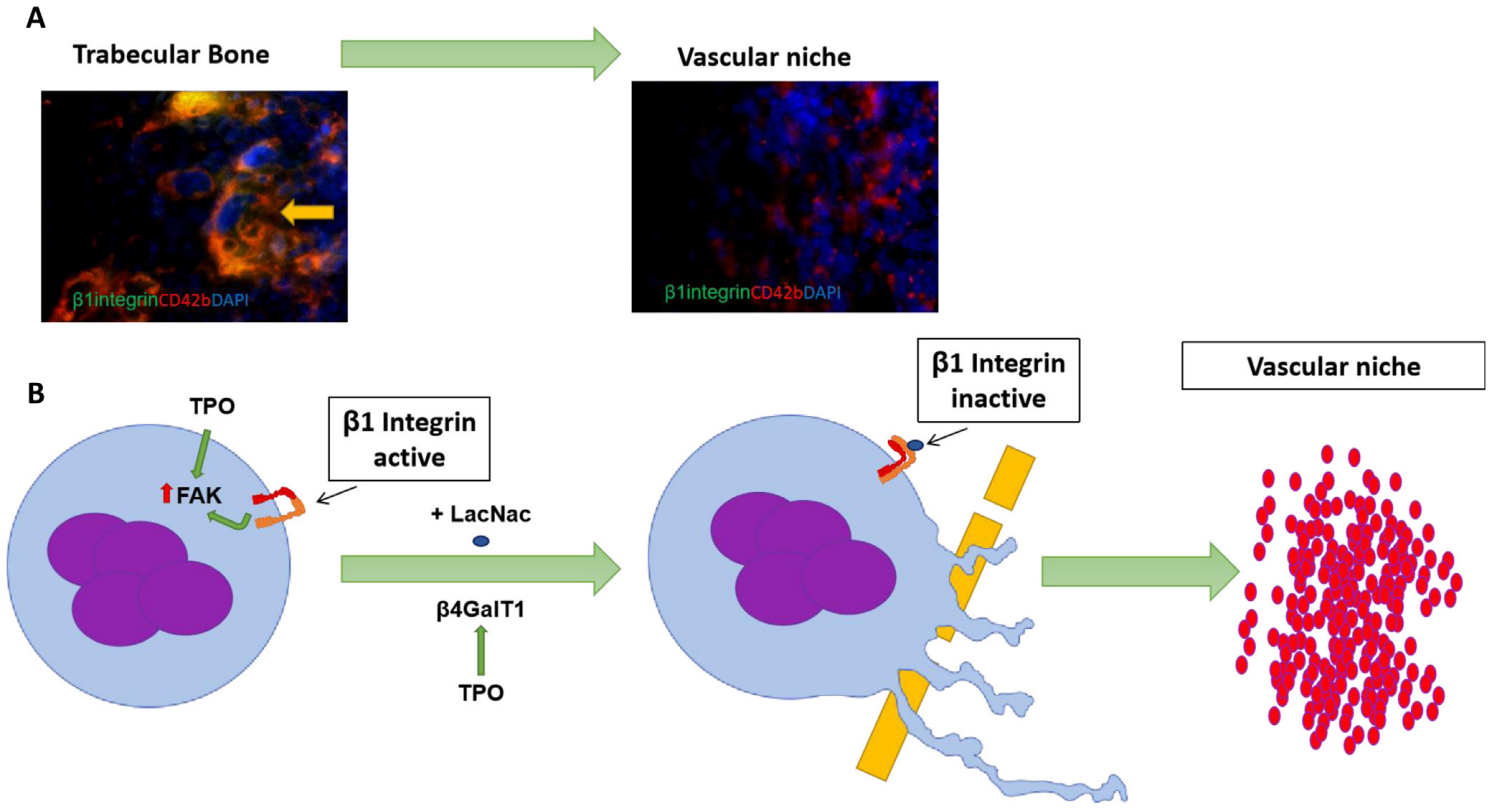
Possible role of β1 integrin activation in the regulation of the latest stages of megakaryocyte (MK) maturation. (**A**) Confocal microscopy analyses of MKs identified with the CD42b antibody (red) and the antibody E9G7 (green), that recognizes the active form of β1 integrin, of MKs localized in the trabecular (left) or in the perivascular (right) zone of a mouse femur. Although MKs in the perivascular niche express β1 integrin (not shown), the protein is mostly inactive, as demonstrated by the absence of green fluorescence signal on the MK in the right panel. By contrast, MKs present in the trabecular bone express robust levels of active β1 integrin, as indicated by the high level of orange (red plus green) signal, and therefore are presumably firmly bound to the extracellular matrix. Nuclei are counterstained with DAPI (blue fluorescence signal). Original magnification 60×. (**B**) In immature MKs, β1 integrin is active and the MK is retained in the trabecular bone. During maturation, the N-terminal domain of the active β1 integrin is glycosylated (blue oval) by the enzyme β4-galactosyl transferase 1 (β4GalT1), inhibiting the binding of the complex to the extracellular matrix and allowing the cells to migrate to the endosteal niche, where they engage the endothelium to release platelets. Thrombopoietin (TPO) and CXCL12 indirectly potentiate both the signal downstream to the active β1 integrin—by increasing the expression of focal adhesion kinase (FAK)—and the level of β1 integrin inactivation, by increasing the expression of β4-galactosyl transferase 1. See 55 and “The role of integrins in thrombopoiesis” section of this article for further detail.

Recently, Giannini and colleagues^55^ identified an additional mechanism that allows MKs to leave their endosteal niche to reach the endothelial niche where they produce platelets (Figure 3). These authors first identified that β1 integrin is a substrate for the enzyme β-1,4-galactosyltransferase encoded by the *β4Galt1* gene localized on human chromosome 9q13. This gene is altered in one of the classes of congenital disorders of glycosylation, in one case of Dandy–Walker syndrome, and in severe inherited neurological malformations^56^. All of these disorders are associated with thrombocytopenia. β4Galt1 is a type II membrane protein localized in the Golgi and on the plasma membrane that catalyzes the addition of β-galactose to the N-acetylglucosamine residues of numerous glycoproteins. In MKs, the substrates of β4Galt1 include β1 integrin and the expression of this protein is upregulated by TPO and CXCL12. Using loss-of-function *β4Galt1* animal models (*β4Galt1*^−/−^ mice), these authors then observed that the HSCs of these mice are biased toward myeloid differentiation and have limited MK differentiation potential. In addition, their MKs are retained at greater frequency in the endosteal niche and have an immature morphology which includes reduced DMS and limited platelet territories. The few platelets present in their bloodstream, however, are functionally normal. The fact that the phenotype of *β4Galt1*^−/−^ mice is completely rescued by MK-specific deletion of *ITGB1* provides final proof that β4Galt1-dependent inactivation of β1 integrin plays a determining role in terminal MK maturation. In conclusion, these results indicate that activation of β1 integrin is required for the first steps of terminal MK maturation supported by the endosteal niche but that its chemical inactivation by β4Galt1-dependent galactosylation is required for the migration of these cells to the vascular niche to complete their maturation (Figure 3). It should be noted that, although both steps are indirectly regulated by TPO (and CXCL12), through upregulation of FAK content first and of *β4Galt1* content later, the primary outside-in signal that regulates β1 integrin function in MKs has yet to be identified.

β1 integrin is also necessary to maintain the homeostasis of the vascular niche. In fact, β1 integrin is expressed on the plasma membrane of the endothelial cells and, by sensing the unidirectional forces of the blood flow, activates the signaling that regulates the alignment of these cells along the vascular walls^57^. This observation opens the possibility that β1 integrin coordinates the anatomical relationship between the MKs and the endothelial cells at the site of platelet formation.

Platelets express the α2b, α5, and α6 and β1 and β3 integrin subunits on their plasma membrane^58^. In particular, β1 may form heterodimers with α2b, α5, and α6. Each of the resulting complexes binds to a specific protein of the ECM: α2β1 binds collagen, α5β1 binds fibronectin, and α6β1 binds laminin^58^. Loss-of-function studies in mice indicate that complexes, including the β1 integrin, may represent the receptors which signal the secretion of the platelet granules that triggers the hemostasis process^59^.

The most studied of the platelet integrin complexes, however, is αIIbβ3, which is expressed in cells at all stages of terminal MK maturation^60^ (Figure 1). This complex in involved in the platelet activation process during hemostasis^61^. In particular, the β3 subunit of this complex triggers platelet pro-coagulation activity by exposing the phospholipid phosphatidylserine that allows granules with pro-coagulation factors to release their content^62^. Activation of β3 integrin is mediated by G protein signaling and promotes binding of talin 1 and kindlin 3 to the β3 integrin tail, providing the inside-out signaling that induces a conformational change of the extracellular domain of the complex that increases its affinity for fibrinogen (in addition to α2bβ1, αIIbβ3 also binds fibrinogen) and for vWF, the first element of the coagulation cascade^63,64^. The fundamental role played by β3 integrin in triggering coagulation has clinical significance since constitutively activation of β3 integrin leads to macro-thrombocytopenia and bleeding disorders in patients with Glanzmann thrombasthenia^65^, suggesting that pharmaceutical inhibition of this subunit may represent a useful antithrombotic strategy to reduce thrombus formation yet avoid the side effect of excessive bleeding that characterizes many of the drugs targeting other components of the coagulation cascade.

In summary, although integrin complexes containing the β1 and β3 subunit are expressed at all stages of terminal MK maturation, it appears that complexes containing the β1 subunit regulate mostly the migration of the cells within the marrow architecture but that those containing the β3 subunit control the response of platelets to hemostatic challenges. However, emerging evidence indicates that, in addition to exerting homeostatic functions depicted in Figure 1, MKs may play important roles as shapers of the containing microenvironment and of the immune response^1,66^. Therefore, we foresee that new studies will soon provide novel information on the regulation exerted by integrins on these additional MK functions.

## Role of integrins in the activation of transforming growth factor beta

In addition to regulating the spatial location of the MKs in the marrow microenvironment, integrins control TGF-β activity^67^. Although TGF-β is a pro-inflammatory cytokine produced by many cell types, MKs are the richest source of the cytokine^68,69^. TGF-β is translated as a propeptide, which is cleaved in the Golgi into three polypeptides: the active TGF-β peptide, the latency-associated peptide–TGF-β polypeptide (LAP–TGF-β), and the latent TGF-β–binding protein (LTBP). TGF-β may be secreted by the cells as a small latency complex (SLC), a soluble trimer formed by one active TGF-β polypeptide encased (and inactivated) by non-covalently binding within two LAP–TGF-β. Alternatively, TGF-β can be secreted as a microenvironment-specific large latent complex (LLC) in which one of the LAP–TGF-β is covalently bound to LTBP, which anchors the LLC to elements of the ECM^67^ (in particular, to collagen^70^). On demand, the conformations of SLC and LLC are modified by either proteolytic enzymes (SLC) or by factors present on the surface of the cells that bind the LTBP tail (LLC), and the active TGF-β polypeptide is released to exert its biological functions^67^. A group of proteins capable of binding to LTBP and activating TGF-β includes the β subunits of integrins, some of which, like integrin β3 and possibly β1, are expressed by the MKs^67,71,72^. Robust evidence indicates that TGF-β elicits a SMAD5 signaling that retains MK immaturity^73^ and promotes their cell fusion and endomitosis^74^. Therefore, the possibility exists that in addition to activating other cells (such as HSCs and osteoclasts)^75^, the SLC released by the MK, once activated by the β integrins on their cell surface, regulates their maturation in an autocrine fashion. The role exerted by integrins expressed by platelets in the activation of TGF-β in the pathogenesis of cancer and other diseases is discussed in more detail in the section below. As much as β1 and β3 integrin may activate TGF-β1, TGF-β1 reciprocally influences the expression of these two subunits^76,77^. It has been demonstrated, for example, that the podocytes of the rat glomeruli respond to TGF-β1 by activating a MAPK signaling that regulates the ratio of β1 and β3 integrin expressed by these cells, altering their adhesion and migration properties^78,79^. Whether this regulatory feedback loop also operates in MKs has not yet been established.

## Role of β1 integrin alterations in the etiology of diseases associated with altered megakaryocytopoiesis, including cancer

Numerous studies indicate that alterations in integrin signaling are directly (by supporting survival, proliferation, and metabolic adaptation) and indirectly (by supporting the formation of a tumor-promoting microenvironment) implicated in cancer progression^80^. Some of the microenvironmental defects induced by integrin activation that may promote tumor growth are represented by neo-angiogenesis and inflammation^81^. β1 integrin plays a significant role in cancer progression and therapy resistance in hepatocellular carcinoma^82–84^ by modulating the exit of cancer cells from dormancy, leading to metastatic growth^85^⁠ and increasing their malignant potential in transplantation models^86^. In addition to playing a role in cancer development, activation of β1 integrin plays a major role in the pathology of inflammation^87,88^. In fact, integrins expressed on the surface of leukocytes favor the migration of these cells to inflamed tissues where, once activated by the presence of inflammatory molecules, the leukocytes exert their cytotoxic activities^87,89^.

Alteration in TGF-β signaling is a well-established factor in the etiology of cancer^67,69,90^, and numerous recent reviews have pinpointed the role played by integrins in the activation of TGF-β in the pathogenesis of cancer and other diseases^91–93^. A specific role in TGF-β–mediated cancer progression appears to be exerted by αIIbβ3 integrin expressed by platelets^61^, but a possible role for β1 integrin expressed by MKs has not yet been excluded. These discoveries catalyzed the development of numerous integrin antagonists, and the clinical goal was to delay tumor progression in patients with cancer or reduce the toxicity of inflammation or both^94^. In view of the role exerted by αIIbβ3 in the pathogenesis of cancer, cilengitide^95^, a compound developed by Merck KGaA (Darmstadt, Germany) that inhibits β3 integrin signaling in endothelial cells by binding the arginine–glycine–aspartate tripeptide in its intracellular domain and preventing its interaction with the FAK/Src/AKT pathway, was tested in a clinical phase 3 investigation (that ended in 2014) for glioblastoma (NCT00689221^96^). Antagonists of β1 integrin currently investigated in clinical trials include volociximab^97^, a monoclonal antibody that was developed by PDL BioPharma (Incline Village-Crystal Bay, NV, USA) and Biogen (Cambridge, MA, USA) and that targets α5β1 integrin. The antibody inhibits neo-angiogenesis and cell migration and was tested in a clinical study for overcoming resistance to cytotoxic chemotherapy in advanced non-small cell lung cancer (NCT00654758^98^, phase 1b), renal cell carcinoma (NCT00100685^99^, phase 2), pancreatic cancer (NCT00401570^100^, phase 2), and melanoma (NCT00099970^101^, phase 2). GLPG0187^102^ is an integrin receptor antagonist that was designed by Galapagos SASU (Romainville, France) and that inhibits α5β3 but is also active in complexes formed by β1 integrin. GLPG0187 suppresses expression of Snail1, Snail2, and Twist (E-cadherin suppressors), decreasing the adhesion and migration of human prostate cancer cells. In murine models, this compound inhibits progression of bone and visceral metastasis and is being tested in a clinical phase 1 study in patients with end-stage cancer (NCT01313598^103^). ATN-161 is a small peptide that inhibits α5β1 integrin and that, in combination with 5-fluorouracyl, reduces liver metastasis and increases survival in murine models of colon cancer^104^. ATN-161 has been developed by Attenuon, LLC (San Diego, CA, USA) and is being tested in a phase 2 clinical study for advanced solid tumors (NCT00131651^105^). These drugs represent potentially useful compounds for many human diseases and for those associated with MK abnormalities with increased expression of β1 integrin, such as myelofibrosis.

Primary myelofibrosis is the most severe of the Philadelphia-negative myeloproliferative neoplasms^106^. This disease currently represents an unmet clinical need because drugs targeting the driver mutations, such as the JAK inhibitor ruxolitinib, are effective in ameliorating symptoms but it is uncertain whether they are effective in halting disease progression^107^. Myelofibrosis is associated with profound MK abnormalities which have been suggested to drive the disease by promoting a malignant cell–supportive microenvironment^108^. In fact, the MKs of these patients remain immature, display high proliferation rates, and release high levels of TGF-β in the microenvironment, leading to fibrosis and failure of normal hematopoiesis in the bone marrow^25,109–111^. In animal models, it has been shown that JAK2V617F, the most common driver mutation found in myelofibrosis, activates β1 integrin in granulocytes and that this activation favors the adhesion of these cells to the endothelial cells, triggering the high rate of thrombosis found in these diseases^112,113^. The observation that β1 integrin activation is induced by JAK2V617F in granulocytes suggests that this integrin is likely activated in the MKs of these patients as well. In fact, the α5 subunit of the α5β1 integrin complex has been shown to be overexpressed in MKs from myelofibrosis patients and JAK2V617F mouse models and its inhibition rescues the malignant phenotype of these mouse models^114^. However, since α5 integrin has mostly a bystander role in the activity of the complex, this article has not excluded that overexpression of α5 does not affect the activity of the β1 subunit as well. The corollary that β1 integrin activation in MKs may contribute to bone marrow fibrosis is consistent with the causative role exerted by β1 integrin activation as an inducer of fibrosis in other organs, such as liver and lung, in animal models^115,116^. The mechanisms of this fibrosis-promoting effect may be represented by activation of the MK-supporting role of the ECM^117^ or by favoring the release from MKs of TGF-β already in an active configuration or by both^67^. In fact, it is well established that increased expression of TGF-β in the microenvironment may induce fibrosis by activating the fibroblasts to secrete collagen^98^ and by increasing the levels of lysyl-oxidase released by the MKs necessary for its polymerization^118^. This knowledge has suggested the therapeutic hypothesis, currently under clinical investigation, that myelofibrosis can be treated by the TGF-β1–specific trap AVID200 (NCT03895112^119^)^120^. However, the role played by β1 integrin on TGF-β activation discussed above suggests that drugs targeting this integrin subunit are also potentially effective in treating myelofibrosis.

## Conclusions

New knowledge indicates that integrin complexes play a major regulatory role in all stages of thrombocytopoiesis, from lineage commitment to terminal MK maturation and platelet release, as well as in the initial steps of thrombus formation. Based on these important roles, congenital and acquired integrin alterations are associated with several non-malignant and malignant platelet disorders. Given the more general role emerging for these complexes in cancer, numerous drugs targeting integrins have become available. It is conceivable that these drugs will soon be demonstrated effective to treat diseases associated with increased risk of thrombosis or myelofibrosis or both.
